# Retraction

**DOI:** 10.1111/cas.15838

**Published:** 2023-05-11

## Abstract

Retraction: Kim, Areumnuri; Lee, Hyunjung; Shim, Sehwan; Kong, Jun Suk; Kim, Min‐Jung; Park, Sunhoo; Lee, Seung‐Sook, Overexpression of dopamine receptor D2 promotes colorectal cancer progression by activating the β‐catenin/ZEB1 axis, Cancer Science 2021, 112 (9), pp. 3732–3743 (https://onlinelibrary.wiley.com/doi/10.1111/cas.15026).

The above article, published online on 12 June 2021 in Wiley Online Library (wileyonlinelibrary.com) has been retracted by agreement between the journal Editor in Chief Masanori Hatakeyama, Japanese Cancer Association and John Wiley and Sons Australia, Ltd. following an investigation based on concerns on figures 1, 2, 3 and 6 raised by the publisher's image integrity team. The corresponding author asked to retract this manuscript as the experimental data in the article could not be reproduced and original data are no longer available to validate the conclusions. The co‐authors were not available to confirm the retraction.

Retraction: Kim, Areumnuri; Lee, Hyunjung; Shim, Sehwan; Kong, Jun Suk; Kim, Min‐Jung; Park, Sunhoo; Lee, Seung‐Sook, Overexpression of dopamine receptor D2 promotes colorectal cancer progression by activating the β‐catenin/ZEB1 axis, Cancer Science 2021, 112 (9), pp. 3732–3743 (https://onlinelibrary.wiley.com/doi/10.1111/cas.15026).

The above article, published online on 12 June 2021 in Wiley Online Library (wileyonlinelibrary.com) has been retracted by agreement between the journal Editor in Chief Masanori Hatakeyama, Japanese Cancer Association and John Wiley and Sons Australia, Ltd. following an investigation based on concerns on figures 1, 2, 3 and 6 raised by the publisher's image integrity team. The corresponding author asked to retract this manuscript as the experimental data in the article could not be reproduced and original data are no longer available to validate the conclusions. The co‐authors were not available to confirm the retraction.

